# Jugular Vein Catheter Design and Cocaine Self-Administration Using Mice: A Comprehensive Method

**DOI:** 10.3389/fnbeh.2022.880845

**Published:** 2022-06-15

**Authors:** Gia Valles, Jessica L. Huebschman, Elsbeth Chow, Corinne Kelly, Yuhong Guo, Laura N. Smith

**Affiliations:** ^1^Department of Neuroscience and Experimental Therapeutics, Texas A&M University Health Science Center, Bryan, TX, United States; ^2^Texas A&M Institute for Neuroscience, Texas A&M University, College Station, TX, United States

**Keywords:** intravenous, drug, self-administration, catheter, operant conditioning, addiction, mouse, jugular vein

## Abstract

Intravenous self-administration (IVSA) is a behavioral method of voluntary drug intake in animal models which is used to study the reinforcing effects of drugs of abuse. It is considered to have greater face validity in the study of substance use and abuse than other assays, and thus, allows for valuable insight into the neurobiological basis of addiction, and the development of substance abuse disorders. The technique typically involves surgically inserting a catheter into the jugular vein, which enables the infusion of drug solution after the performance of a desired operant behavior. Two nose- poke ports or levers are offered as manipulanda and are randomly assigned as active (reinforced) or inactive (non-reinforced) to allow for the examination of discrimination in the assessment of learning. Here, we describe our methodological approach to this assay in a mouse model, including construction and surgical implantation of a jugular vein catheter, set up of operant chambers, and considerations during each phase of the operant task.

## 1. Introduction

Drug intravenous self-administration (IVSA) is a behavioral test that has become widely used and accepted in the study of the reinforcing effects of drugs of abuse using animal models, including rats, non-human primates, cats, and mice. It offers valuable insight into the neurobiological basis of addiction by mimicking real-world behaviors that are associated with recurrent drug use during the development and maintenance of substance abuse disorders. Drug IVSA involves surgically inserting an intravenous catheter, typically into the jugular vein, and uses the principles of operant conditioning, such that the subject learns to perform a desired behavior to receive drug delivery (Panlilio and Goldberg, 2007). Typically, the drug IVSA test is conducted in operant chambers that offer two identical manipulanda—items in the conditioning chamber that can be manipulated physically—such as nose poke ports or levers. Manipulanda are assigned as active (reinforced) and inactive (non-reinforced) to allow both for the analysis of port discrimination in the assessment of learning, as well as for general activity levels. A reinforcement schedule allows the experimenter to modify task requirements for the type of research being conducted (Platt and Rowlett, 2012).

Since IVSA allows for volitional control of intake, it is considered to have higher face validity for addictive behavior than other types of tests, and has been used, for example, to verify that nicotine mediates reinforcement *via* activity at nicotinic acetylcholine receptors (Fowler and Kenny, 2011). An alternative method is oral self-administration, which can be conducted in both two-bottle choice paradigms, as well as operant paradigms; however, there are trade-offs to consider for the two methods (refer to Table 1). Advantages of the IVSA method include allowing the experimenter to remove possible confounding variables and accurate quantification of the amount of drug consumed. Along with increased accuracy, the IVSA allows for a rapid increase in drug levels in the blood and brain (Kmiotek et al., 2012). The IVSA is also the gold-standard test for assessing the addictive propensity of novel compounds, and the significance of prior drug exposure can be investigated. In addition, infusions can be paired with discriminative cues to allow for later evaluation of drug-seeking behaviors following re-exposure to a reinforcer-paired cue (Thomsen and Caine, 2005), which is often used as a model of relapse (Galaj et al., 2016). Abstinence, or time without access to a drug, causes a deprivation effect and can be used to assess drug-seeking (when no drug is available) or changes in administration upon renewed drug access. For example, “incubation” of craving has been demonstrated for cocaine, where cocaine-seeking escalates between 1 and 45 days of abstinence following prior repeated exposure.

**Table 1:** Considerations for choosing intravenous vs. oral routes of drug self-administration.

	**Advantages and disadvantages of intravenous vs. oral drug self-administration models**
Advantages of IVSA	• Onset of drug action for IVSA is more rapid than oral SA, and thus, better mimics certain types of human drug abuse. • IVSA allows more assurance, as well as accurate determination of the volume, of substance delivery. • IVSA allows higher bioavailability of substances, bypassing the need for absorption through the digestive tract, avoiding digestion-related substance degradation, and avoiding first-pass metabolism by the liver. • IVSA eliminates intake alterations or avoidance related to taste.
Disadvantages of IVSA	• IVSA is less convenient and safe than oral SA, necessitating major surgery and introducing opportunities for infection. • IVSA is less economical, requiring single-use catheters and surgical materials. • IVSA has lower throughput than oral SA due to loss of catheter patency and procedure-related mortality in a subset of animals. • IVSA requires the continued presence of invasive materials in/on the body and catheters must be maintained by regular flushing. • IVSA always requires some level of active training/learning, whereas some forms of oral self-administration (e.g., two-bottle choice) are minimally dependent on such processes.

Using the drug IV self-administration assay, many factors, both intrinsic and extrinsic, have been shown to influence voluntary drug-taking. Intrinsic factors, such as exploratory and risk-taking behaviors in mice, are associated with an increased propensity to self-administer cocaine (Dickson et al., 2016). Sex is also an important factor, since female rats self-administer more nicotine, for example, than male rats (Galankin et al., 2010; Flores et al., 2019). Extrinsic factors, such as social learning and environment, also influence the tendency of an animal to self-administer drugs. A study in which rats had to drink a saccharin solution to get infusions of nicotine revealed that interaction with another rat drinking the solution increased the likelihood of stable drug-taking behavior (Wang et al., 2016). There are mixed findings on the impact of diet, with studies showing that high-energy diets either do not change (Bruggeman et al., 2011) or suppress cocaine reinforcement (Wellman et al., 2007). The IVSA has been shown to be reduced following exercise, with rats reducing methamphetamine intake after just 1 day of access to an exercise wheel (Aarde et al., 2015). The acquisition of the drug IVSA in rats may also be elevated by higher ambient temperature (Aarde et al., 2017). In comparison to basic laboratory animal housing, enrichments, such as toys, obstacles, and running wheels decrease the drug self-administration (Ewing and Ranaldi, 2018). These findings have broad implications for the types of questions that can be answered using this important behavioral technique.

Rodents are often used in IVSA studies, but there exists a historical preference for using rats over mice, likely due to feasibility (refer to Table 2 for a comparison of the two species). In rats, the earliest veinous catheters for drug delivery emerged by the early 1960s (Slusher and Browning, 1961; Weeks, 1962), while the first mouse catheters were described nearly 20 years later (Barr et al., 1979). However, self-administration of substances in mice first relied on tail-vein injection without catheters (Criswell, 1982; Criswell and Ridings, 1983), and mouse self-administration using chronic indwelling catheters was only described later (Carney et al., 1991; Grahame et al., 1995; Deroche et al., 1997). However, with the development of new and smaller materials and model-related motives for using mice to test the role of genetics in substance abuse, more labs are attempting mouse IVSA. In the current methodological description, founded originally on methods developed by Thomsen and Caine (2005), we focus on cocaine IVSA using a mouse to model and measure the addiction-related behavior. Our primary goals in this manuscript are to increase the transparency of methodological choices in mouse IVSA, and likewise, increase the accessibility of the technique. We describe the surgical procedure we use and demonstrate and explain the importance of different phases of the behavioral test, including acquisition, extinction, measurement of a dose-response curve, and increasing the cost. We also discuss other methodological approaches that may be selected for different purposes.

**Table 2:** Considerations for mouse intravenous self-administration compared to other methods.

	**Comparison of mice and rats for use in IVSA**
Basic study logistics	• Using mouse models allows leverage of a vast array of genetic models. • Mice typically have cheaper housing facility costs. • Smaller testing equipment for mice allows more to fit into a lab space. • Mice may be less destructive to some materials and equipment.
Surgical outcomes	• Rats have larger anatomical structures, which facilitate surgery and aide catheter patency, but the smaller anatomy of mice can be offset by thoughtful catheter choices and patient, observant surgical practice. • Greater irritation and rupture of skin around the catheter base in mice can be significantly reduced by using catheters with highly pliable (e.g., monofilament polypropylene) surgical mesh. • Rats may have lower surgery-associated mortality by conventional methods, but survival is improved in mice by using sevoflurane, which speeds recovery from anesthesia. Special attention to humidity and hydration also improves outcomes in mice (Thomsen and Caine, 2005).
Task feasibility	• Ratsare credited with ability to perform more complex tasks than mice; however, we find mice capable of tasks sometimes labeled as “too difficult” for them (e.g., reinstatement of cocaine IVSA, increased schedules of reinforcement). Being natural prey animals, fear management (e.g., preparatory handling sessions prior to procedures, calm experimenters, test room acclimation) is essential in mice.

## 2. Materials And Equipment

### 2.1. Catheter Assembly

#### 2.1.1. Prepare Tubing-Attached Syringes

**Figure 1:**
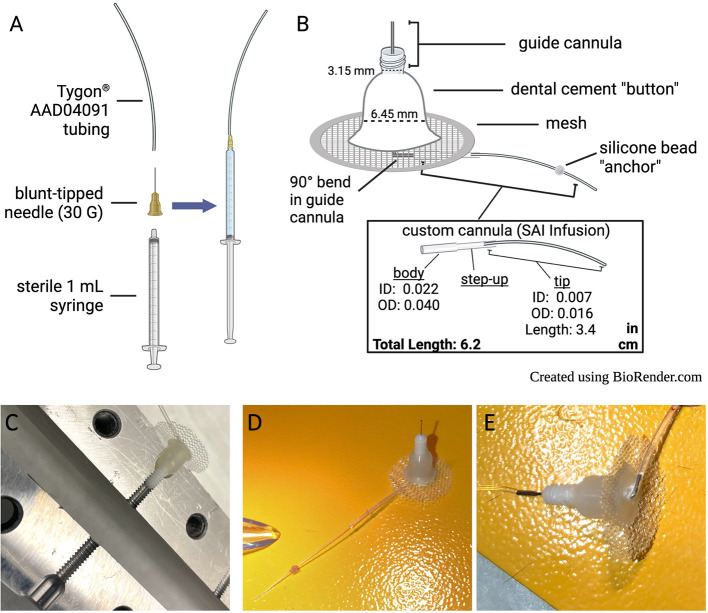
Catheter design and care. **(A)** Set-up for attaching tubing to syringe for surgical drug delivery, catheter flushing, and checking catheter patency. **(B)** Descriptions of catheter components. **(C)** Custom acrylic base mold, in open position, with completed catheter in place to demonstrate mold placement. **(D)** Picture of assembled catheter. **(E)** Underside of assembled catheter.

2.1.1.1 All purchased materials and manufacturers are listed in Table 3. Details and images of an assembled catheter can be seen in Figure 1, and tips for troubleshooting surgery and behavioral testing can be found in Table 4.
2.1.1.2 Cap 1 mL syringe with blunt-tipped needle and attach tubing to the end as shown in Figure 1A. Tubing-attached syringes will be used for checking catheter patency, surgical drug delivery, and catheter flushing, so it may be useful to prepare multiple syringes.

**Table 3:** Description of materials used in the protocol.

**Description in protocol**	**Catalog name of material/equipment**	**Company**	**Catalog number**
**Catheter assembly**			
1 mL syringe	1 mL Syringe	VWR	BD309659
Blunt-tipped needle	Sterile Blunt Needles, 30 Gauge, 0.5-inch Length	SAI Infusion Technologies	B30-50
Tubing (for syringes, drug delivery lines, catheter caps)	Tygon® non-DEHP Medical Microbore Tubing, 0.010*″* ID × 0.030*″* OD ND-100-80	SAINT-GOBAIN PPL	AAD04091
Custom cannula/catheter tubing	Mouse Jugular Vein Catheter	SAI Infusion Technologies	MJC-21
Guide cannula	C315G—ICV Single Guide Cannula, 26 Gauge Stainless-Steel, Short Pedestal (5 mm UP), cut 10 mm below pedestal	P1 Technologies	81C315G5UPSC
Super glue	Loctite® Professional Super Glue	LOCTITE	500041-008
Monofilament polypropylene mesh	Premilene Mesh 26 cm × 36 cm	B Braun Surgical	J1249C
Arch punching tool	General Tools® 1271-334-−3-3/4" Arch Punch	General Tools	1271-334
Custom catheter base mold	custom machined using catheter base and guide cannula specifications	See Figure 1C	N/A
Mold release	3-IN-ONE 4-oz All-temperature Silicone Drip Oil	WD-40 company	3IO-SIL-00
BCA liquid	Ortho-Jet BCA Liquid	Lang	B1303
BCA powder	Ortho-Jet BCA Powder	Lang	B1320
Silicone	Aquarium-Safe Silicone	GE	Rev0917
**Juglar vein catheter implantation surgery**			
21G winged needle (for catheter sled)	SURFLO Winged Infusion Set	TERUMO	350761071
Artery scissors	Bonn Artery Scissors-Ball Tip	FST	14086-09
Fine scissors	Hardened Fine Scissors (24 mm cutting edge; length 9 cm)	FST	14090-09
Curved forceps	Dumont #7 Forceps-Standard/Dumostar	FST	11297-00
Curved hemostats	Kelly Hemostat	FST	13019-14
Straight hemostats	Kelly Hemostat	FST	13018-14
Surgical bar	Metal bar, ~1–2 mm diameter; ~10 cm length	See Figures 4D,E	N/A
Cefazolin	Cefazolin sodium, preservative free	WG Critical Care	NDC 44567 707
Heparin	Heparin Sodium Injection	SAGENT	49130
Saline	Sodium Chloride Injection, USP, preservative-free, 0.9% Solution	Covetrus	009861
Ketoprofen	Ketofen, 100 mg/mL	Zoetis	005487
Sevoflurane	Sevoflurane, USP	Covetrus	035189
Anesthetic vaporizer	Somno Suite Low-Flow Anesthesia System	Kent Scientific	ss-01
Gas delivery nose cone	Anesthesia Masks/Breathing Circuits for SomnoSuite®	Kent Scientific	SOMNO-0305
Surgery platform	QuadHands Workbench—Helping Hands Third Arm Soldering Work Station w/steel base and 4 flexible magnetic arms	QuadHands	QH-WB-DELUXE
LED 3X magnifier	QuadHands LED 3X Magnifier with Rare Earth Magnetic Base	QuadHands	B078MWYRCH
Magnetic twist ties	TwistieMag Strong Magnetic Twist Ties	Monster Magnetics	B07V5H5X8K
Hair trimmer	Mustache & Beard Battery Trimmer	WAHL	Model 5606
Eye lubricating ointment	Artificial Tears	HENRY SCHEIN	048272
Triple antibiotic cream	Triple Antibiotic Ointment	Acme United Corporation	76049-190
Synthetic absorbable sutures	coated vicryl synthetic absorbable sutures 4-0/SA SH-1/27 IN	Ethicon	J310H
Metal dust caps (thread must match guide cannula on catheter)	Round, standoff, aluminum, female-female, 3/4 in overall length	GRAINGER	6MZE4
**Operant conditioning boxes**			
Operant boxes	Habitest Modular Test System—Mouse, including test cage, wall panels, house and cue lights, nose poke ports, flooring, power base and control board, counter-balance arm	Coulbourn	H10-11M-TC, H01-01, H02-08, H03-04, H90-00M-KT01, H11-01M-LED, H10-11M-TC-SF, H21-09M, H11-03M-LED, H20-94, H29-01
Light-attenuated chamber	Isolation Cubicle, Tall	Coulbourn	H10-24T
Syringe pump	Programmable Speed Infusion Pump	Coulbourn	E73-02
Swivel	Mouse Swivel	SAI Infusion Technologies	A150140
Tether	Spring Tether with 6–32 Threaded End, 15*″* Length	SAI Infusion Technologies	TT-15 (may need to request)

**Table 4:** Troubleshooting, tips, and tricks.

	**Problem/question**	**Solution, tip, or trick**
During surgery and recovery	Anesthesia cone will not stay properly positioned.	Magnetic twist ties can be used to elevate and help secure the anesthesia nose cone.
	The mouse jugular vein cannot be located.	The pectoral muscle lays like a pink blanket tucking in the jugular vein, which peeks out from the top. Once the skin incision is made midway between sternum and the ear, use forceps to gently slide top layers of tissue toward the *animal's* left side, which often reveals the dark red vein. If not successful, use forceps to break the overlying clear fascia, then repeat above. If not successful, puncture the visible layer of tissue discretely and continue to pull aside top layers as you look for the vein.
	While clearing tissue from the jugular vein, the vein tears.	Immediately staunch the bleeding with firm but gentle pressure to the vein or by lifting the surgical bar. If the bar is not in place, try to make a small space to insert it while continuing to control any bleeding. It is possible to place a catheter in a torn vein if it is not severed. To avoid tearing the vein, remove fatty tissue by gripping it away from the vein and slowly pulling in a motion parallel to the vein (not perpendicular). Do not grip the vein directly or pull the clear surface of the vein. Opaque tissue fibers that remain close to the vein can be separated and cut against the surgical bar.
	Death occurs when using catheter sled.	Vulnerability to this issue differs by mouse size and background strain. The sled should be inserted minimally and not more than 5 mm. A stack of gauze near the mouse's head should be used to keep the winged end of the sled propped at the level of the vein or slightly above.
	The catheter cannot be fully pushed in or pushes back out. AND/OR No blood is seen at pullback and pulling the catheter out halfway does not fix this.	The catheter is inserted between the vein wall and a sheathe that surrounds the vein. Double check that the vein cut resulted in bleeding. If not, or if there is a tiny amount of blood, make a slightly larger cut at the same position. If bleeding indicates the cut is already adequate, shifting the catheter (or sled) introduction point up or down the vein length from the perceived cut can help.
	Attempts at vein entry are not successful.	Minimize manipulation of the vein at every opportunity, as it tends to shrink the vein. Target the catheter (or sled) to enter the vein slightly higher or lower than the perceived cut. Check whether bleeding indicates a sufficient cut and consider improving the cut. If the sled has not been used, try it. If too much time has passed, blood may have clotted. Use rinsing syringe of saline to clear and moisten vein. If saline does not help, a new cut may be required.
	Surgery time and mortality need to be reduced.	Improve technique by inserting the catheter without a sled. While cutting the vein, look closely for the exit point of blood, then move swiftly to introduce the catheter tip firmly at that site, using a lateral and downward motion (toward the mouse's vein and feet). If not successful, a sled can still be used.
	Blood was flowing with syringe pullback, but now there is no blood flow with pullback.	Vein suture ties may be too tight. Check blood flow just after securing the ties. If flow is diminished, minimally loosen the ties one-at-a-time and recheck, repeating until flow is restored. If blood flow was confirmed after vein sutures were tied but stops after the mouse is supine, the tubing may be constrained. From the back incision, seat the catheter base and slightly rotate it left or right to adjust the direction of the tubing as it exits the base, while checking blood flow. If blood remained visible in the tubing after a blood flow check, it may have coagulated. Ideally, pushing a tiny volume of saline into the line should move blood easily. If not, gently alternating between pushing and pulling the syringe plunger may loosen the clotting. Avoid pushing much saline into the animal as it can result in death. If blood flow cannot be confirmed before ending surgery, the back and/or chest incisions will need to be reopened, and tubing direction, vein sutures, and catheter entry reexamined, checking for blood flow until it has returned.
	Mice die or become ill during recovery.	Give post-surgery and recovery administrations of USP-grade saline (0.5–1.0 mL per mouse, s.c., 1–3 times/day). Separate mice recovering from anesthesia from cage mates not anesthetized for at least 24 h. If mice become ill despite antibiotic use, ask your facility vet to test for presence of harmful, systemic bacteria and advice on antibiotic choice.
	During operant conditioning	Mice are slow to acquire task.	Ensure mice are not sitting in chambers for long after sessions have ended. Some or all these interventions can be used: vanilla flavored Ensure® around/on active manipulandum; 1–3 non-contingent (priming) drug infusions (depending on concentration) either at session beginning or ~15 min into session; increased drug concentration for 1 session; 1–3 overnight sessions (lengthen session and use timeout periods to minimize overdose risk). Check catheter is patent. If using levers, consider changing to nose poke ports.
Should timeout periods or response maximums be used?		Timeout periods following drug delivery and limitations on the maximum number of responses allowed in a session may restrict the normal range of drug-taking behavior. Consider only using timeouts >3 s for very high doses or when switching from a long period of low-moderate dose availability to a much higher dose.
Mice are slow to extinguish.		Sessions may be lengthened to 5–6 h temporarily. If extinction sessions include the discriminative cue (reinstatement not planned), then response requirements can be increased temporarily (i.e., FR3 or above).
Catheter malfunction or loss of catheter patency during experiment.		Remove the catheter, implant a new one into the jugular vein on the mouse's left side, and allow recovery. If the study uses criteria to determine completion of each phase, run each phase as before, repeating any that were already run. Data from phases obtained originally with a patent catheter should be kept, but use “recath” data for any phases not completed with a patent catheter in the original run. *Note: this approach may not be appropriate for all study designs. Before beginning, plan how to appropriately combine each animal's original and post-“recath” data depending on study goals and design*.

#### 2.1.2. Prepare Catheter Base With Custom Tubing

2.1.2.1 Slide custom cannula over the longer (10 mm) guide cannula end, which will exit the bottom of the catheter base (refer to Figure 1B).
2.1.2.2 Bend the 10 mm metal guide cannula into a right angle where it meets the body of the custom cannula tubing.
2.1.2.3 Secure the custom tubing with super glue. Allow to cure overnight. Glue must be fully cured for further steps.

#### 2.1.3. Check Partially Assembled Catheter for Leaks

2.1.3.1 Place a kimwipe on a flat surface. Make sure to perform any leak checks over the kimwipe so that the source of any leaks can be identified.
2.1.3.2 Press the tip of the finger to the free end of the cannula tubing to prevent water from flowing. Use a tubing-attached syringe to push distilled water through the partially assembled catheter. If there are no leaks, release the finger closing the tube, and ensure that liquid can flow through freely.

#### 2.1.4. Prepare Mesh

2.1.4.1 Cut ~3/4″ circles of monofilament polypropylene surgical mesh using an arch punch (1 per catheter to assemble).
2.1.4.2 Make a 2 mm incision in the center of each mesh circle.

#### 2.1.5. Prepare Acrylic Resin and Assemble Catheter

2.1.5.1 Note: Preparations of the catheter base mold and acrylic resin should be performed in a fume hood. Catheters can be removed from the hood after the curing step.
2.1.5.2 Open custom catheter base mold (Figure 1C). Dip a cotton-tipped applicator in mold release and apply to the inside of each receptacle in the catheter base mold.
2.1.5.3 Place the guide into the catheter base mold with attached tubing facing upwards (i.e., pointed away from the catheter base mold). Close the catheter base mold and tighten it with a screw.
2.1.5.4 In a small petri dish, pour approximately 1:1 volumes of Ortho-Jet BCA powder and liquid.
2.1.5.5 Stir until powder is fully dissolved. The desired consistency is a thin, sticky gel.
2.1.5.6 Fill each catheter base mold receptacle with the BCA mix until it reaches the level with the top of the mold. Be careful not to over or underfill.
2.1.5.7 Slide the catheter base with custom tubing through the incision in a prepared mesh circle. Place the mesh all the way down against the surface of the mold so that it touches the BCA mixture. Make sure that the mesh is centered around the cannula base.
2.1.5.8 Apply a small amount of the BCA mixture to the center of the mesh to seal it against the mixture in the catheter base mold. Make sure that the exposed surface of the BCA mixture is smooth.
2.1.5.9 Allow the BCA mixture to cure overnight, then remove catheters from the catheter base mold.
2.1.5.10 Check catheter for leaks (repeat step 2.1.3).

#### 2.1.6. Finish Catheter Construction

2.1.6.1 Measure 4.5 cm of catheter tubing starting from the center of the guide (where the metal is attached to the plastic). Use a permanent marker to mark the spot.
2.1.6.2 Apply a small ball of silicone at the mark, making sure to fully encompass the diameter of the tube. Allow the silicone to cure overnight.
2.1.6.3 Measure from the silicone ball and use a scalpel blade to shorten the end of the catheter with a slightly beveled cut to 1.2 cm long^1^.
2.1.6.4 Check catheter for leaks (repeat step 2.1.3).
2.1.6.5 Store trimmed catheters (see Figures 1D,E) in a closed, clean container until surgery.

#### 2.1.7. Make Plastic Catheter Caps

2.1.7.1 Cut Tygon® (AAD04091) tubing into 4 mm pieces.
2.1.7.2 Hold each piece with hemostats at one end and melt the other end in a gas flame. Clamp the melted end quickly with another pair of hemostats to seal.
2.1.7.3 After cooling, push caps onto extra “dummy” cannula guides to stretch, so they will be easier to apply to catheters later.

### 2.2. Materials, Instruments, and Drug Solutions for Surgeries (Prepare Ahead of Time)

#### 2.2.1. Make Reusable Catheter “Sled”

2.2.1.1 Place an uncapped 21G winged needle on a flat surface, with the beveled side up. Use a file or sandpaper to extend the bevel further up the shaft, leaving the needlepoint intact. Aim to remove about half the diameter of the shaft for 2 cm in length.

#### 2.2.2. Sterilize Surgical Instruments

2.2.2.1 Sterilize by autoclave, or other acceptable methods, and keep the needles sterile until surgery.

#### 2.2.3. Prepare Cefazolin Aliquots

2.2.3.1 Make several 1.5 mL tubes of 50.25 mg cefazolin powder so they are ready to mix fresh daily into 0.75 mL heparin-saline. Store at room temperature.

#### 2.2.4. Prepare Heparin-Saline Solution

2.2.4.1 To make heparin-saline solution (for the day of surgery and for post-surgery flushing), mix 30 USP units heparin per mL of saline. Keep the solution sterile and make 5 mL of aliquots to be kept at room temperature.

#### 2.2.5. Prepare Ketoprofen Solution

2.2.5.1 Make a dilution of ketoprofen to 1 mg/mL using sterile saline, to be kept in a sealed container. Store at room temperature in accordance with the expiration date on the label.

### 2.3. Operant Conditioning Chamber and Program

#### 2.3.1. Prepare Syringe Pumps

**Figure 2:**
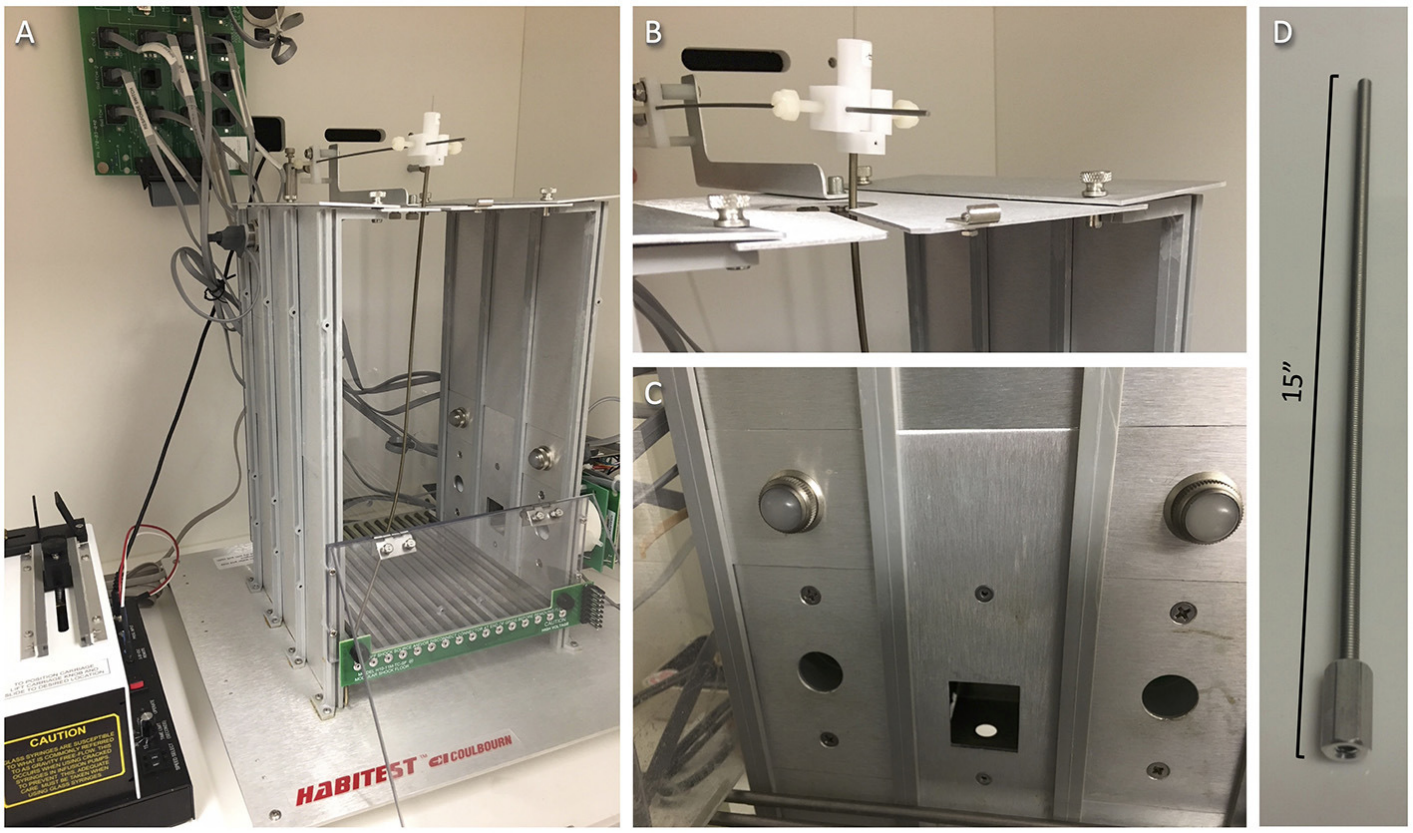
Operant box set-up. **(A)** An example operant box arrangement inside a light-attenuated chamber. Plastic Tygon tubing (not shown) should run down a spring tether (to protect from chewing) and hang ~4 mm outside of the bottom where it can be attached to the mouse guide cannula, then the tether is screwed directly onto the guide cannula threading. At the top, the Tygon tubing should attach to a counterbalanced arm mounted above the chamber using a free-turning swivel (to avoid twisting the line/tether), then connect *via* a blunt-tipped needle to a drug or vehicle syringe (not shown) mounted in the syringe pump (shown left). A house light is located inside the upper left side of the chamber, out of view. **(B)** Closer view of a swivel and counterbalanced arm. **(C)** Closer view of manipulanda (nose poke ports), active and inactive, positioned at the left and right sides of one chamber wall, with visual discriminative cues (cue lights) arranged above each port. **(D)** Closer view of tether with connector matching the catheter guide cannula threading. Tether length may need to be shortened and weight position changed on the counterbalanced arm so that the tether has an ideal amount of slack; it should not pull down the catheter or impede the mouse's ability to move or access all areas of the chamber floor.

**Figure 3:**
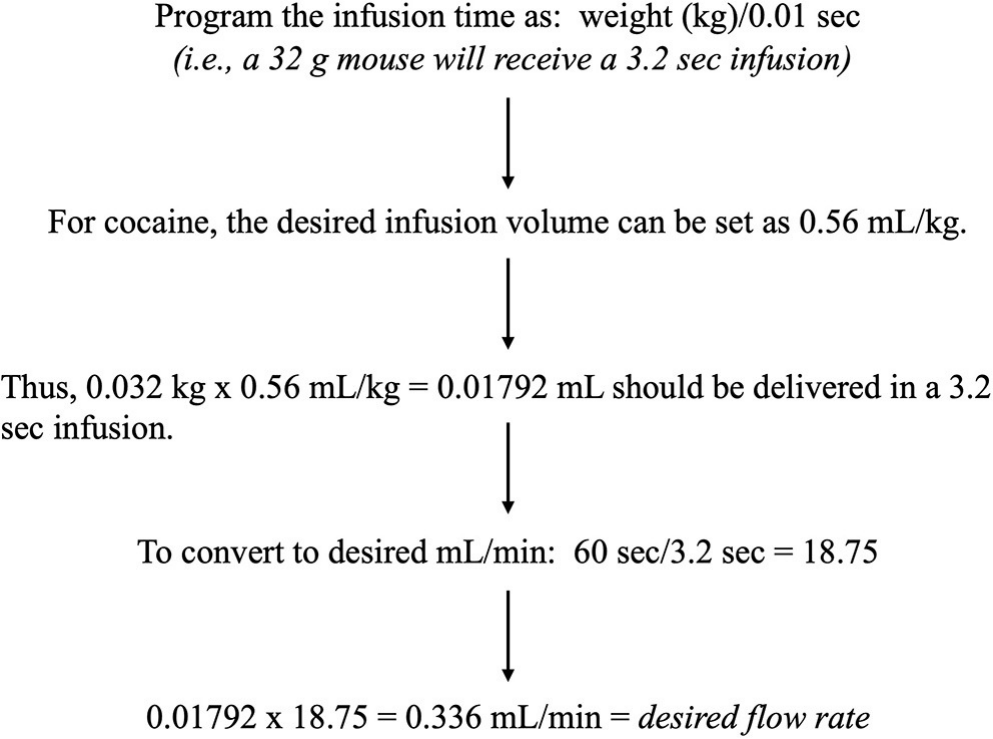
Infusion time calculation. Example formula for infusion time, based on mouse body weight, and desired infusion volume, which are used to calculate the desired flow rate for the set-up of syringe pumps.

2.3.1.1 An example of an operant chamber and details for set-up can be seen in Figure 2.
2.3.1.2 Flow rate (mL/min) must be determined for each syringe pump during the initial setup of IVSA and any time the syringe size is changed. An example of an operant chamber and details for set-up can be seen in Figure 2.
2.3.1.3 Prepare set concentrations of drug according to the desired dose (those used in the current study are available in Table 5) and vary the infusion time according to mouse weight. One option is described in Figure 3.
2.3.1.4 Syringe pump manuals provide an equation with the cross-section area of the syringe^2^ that should be used if it has not already been established.
Example: 0.19538 (known value for E73-02 Model^3^) × 1.3945 (Safety-Lok BD 10 mL cross section area) = 0.272457; 0.272457/0.336 mL/min (desired flow rate) = 1.233 (desired RPM).
2.3.1.5 Now check the syringe pump chart to find the corresponding pump speed setting, which will be applicable for this syringe size.
2.3.1.6 To check the accuracy of calculation or to verify the current pump setting:
2.3.1.5.1 Either weigh two small empty weigh boats and record weights, or plan to check volumes using a P200 pipette.
2.3.1.5.2 Fill the desired syringe type with water and load into the syringe pump, making sure (1) to set the desired pump speed calculated above, (2) to set a mouse weight (e.g., 0.032 kg) in the IVSA program, and (3) when the syringe is primed, then any fluid is cleaned off.
2.3.1.5.3 Using the proper IVSA program and counting infusions, deliver ~3 to one weigh boat, then a larger number (~17) to the other.
2.3.1.5.4 Weigh and subtract the respective starting boat weights for actual fluid weight. For water: 1 g = 1 mL^4^.
2.3.1.5.5 Divide water volume per boat by the number of infusions = volume per infusion. Several measurements should average to the desired volume/infusion (~18 μL in the above example) for the study.

**Table 5:** Cocaine concentrations used in the current study to achieve the per infusion doses listed.

**mg/mL**	**mg/kg/inf**
Saline	0
0.018	0.01
0.056	0.032
0.18	0.10
0.56	0.32
1.8	1.0
5.6	3.2

#### 2.3.2. Program Considerations

2.3.2.1 Operant manipulanda should be randomly assigned as active (provides drug reinforcement) and inactive (no consequence)^5^. Discriminative cues (lights, tones) associated with the active port or lever should be activated with drug delivery. The inactive cue is not used.
2.3.2.2 Timeout periods^6^ are commonly used to slow administration. However, timeouts restrict the number of responses that can be made in a session, and if severe, may limit the ability to observe a normal range of drug-taking behavior. Timeouts may be particularly important when switching from a long period of low-moderate dose availability to a much higher dose^7^. Alternatives to consider may be an intervening step-up dose to allow mice to adjust to the change and/or imposing maximums on allowed responses per session (e.g., 100 responses at doses <1.0 mg/kg/infusion, 30 responses at 3.2 mg/kg/infusion)^8^.
2.3.2.3 In some studies, the house light remains on as long as the program is in session. Alternatively, it may be more specifically used for the signal availability of the drug, remaining on outside of drug delivery and timeout periods.

## 3. Methods

The animal study was reviewed and approved by Texas A&M University Institutional Animal Care and Use Committee.

### 3.1. Jugular Vein Catheter Implantation Surgery

#### 3.1.1. Materials (Prepare Day of Surgery)

3.1.1.1 Set up the gas vaporizer for sevoflurane/oxygen delivery
3.1.1.2 Prepare a rinsing syringe by filling a 5 mL syringe with sterile saline and capping it with a blunt needle.
3.1.1.3 Prepare 1–2 animal hydration syringes (0.5–1 mL, sterile saline) using needles for subcutaneous injection.
3.1.1.4 For the 1 mL tubing-attached syringes (section 2.1.1), load one with the cefazolin/heparin-saline solution and the other with sterile saline. Label each syringe and place them near the surgery area with tips upright to keep sterile.

#### 3.1.2. Prepare the Surgery Area

3.1.2.1 Sterilize the surgery area and, atop the disposable bench pad, arrange the surgical platform, and the required materials, including alcohol pads, drugs, syringes, and instrument tray.
3.1.2.2 Sterilize the surgical platform and secure the anesthesia nose cone or facemask so that the mouse can be placed on the platform in the prone position^9^.
3.1.2.3 Fill the instrument tray with ethanol to ≥ 0.5″, and soak catheters, sealed plastic caps, and metal dust caps.

#### 3.1.3. Prepare the Recovery Area

3.1.3.1 Set a heating pad to the lowest temperature and arrange clean, empty cages for recovery, so they are each sitting half on and half off the heating pad.
3.1.3.2 Each cage should be lined with a clean paper towel.
3.1.3.3 Place the hydration syringes inside one of the cages over the heating pad to warm.

#### 3.1.4. Prepare the Mouse

3.1.4.1 Record the body weight of the mouse.
3.1.4.2 With a hair trimmer nearby, anesthetize the mouse in the induction chamber (7% of sevoflurane in oxygen at ~500 mL/min rate), always maintaining the supervision of the mouse.
3.1.4.3 When breathing is slowed and even, remove the mouse, and shave from just behind the ears to mid-back in a large square. Repeat anesthesia and shaving, if necessary.

#### 3.1.5. Anesthesia Maintenance and Pre-operative Care

3.1.5.1 To begin surgery, induce anesthesia again, then rapidly move the mouse to the face mask or nose cone delivery device and adjust the anesthesia settings as appropriate for the maintenance of anesthesia (2–3% sevoflurane; adjust the flow rate according to vaporizer manufacturer instructions). Monitor the mouse throughout the surgery. If there is gasping, adjust the dial to <2%, and if there is shallow breathing, adjust the dial toward 3%.
3.1.5.2 Next, apply eye lubricating ointment to completely cover each eye. Clean incision sites with an alcohol pad or betadine and allow to air dry. Using an insulin syringe, administer ketoprofen (0.05 mL; subcutaneous) into the lower back.

#### 3.1.6. Position and Prepare Catheter

**Figure 4:**
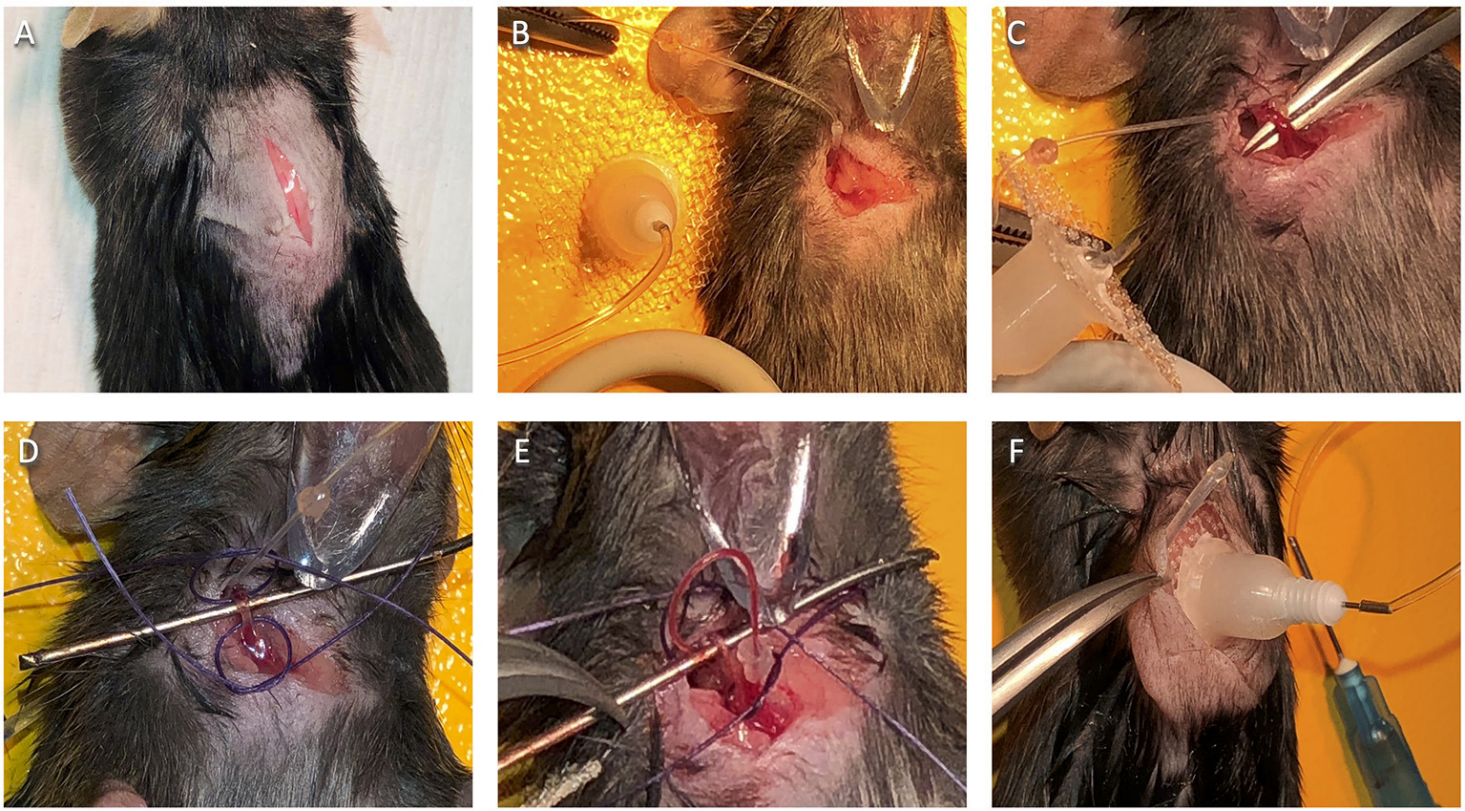
Jugular vein catheter implantation surgery. **(A)** Placement of initial dorsal side incision. **(B)** Catheter placement after successful ventral to dorsal tunnel. **(C)** Isolated jugular vein. **(D)** Jugular vein with suture loops in place, ready for veinous incision. **(E)** Successful catheter placement in the jugular vein. **(F)** Positioning of catheter base on the dorsal side.

3.1.6.1 According to your animal protocol, confirm that the mouse is sufficiently anesthetized. Pull up the skin on the back of the neck using forceps, and at the midline, just behind the base of the ears, create a tiny hole with small surgical scissors. Insert the scissors and cut a ~2 cm incision along the midline of the midscapular region toward the tail (Figure 4A).
3.1.6.2 To make space for the catheter base under the skin, insert the hemostats ~1.5 cm into the incision, making slight opening and closing motions with the tips just beneath the skin in a circular area around the incision. Then, using the same motions with the hemostats, tunnel under the skin over the mouse's right shoulder toward the front of the chest.
3.1.6.3 Turn the mouse to a supine position, moving the face mask or nose cone accordingly^10^.
3.1.6.4 Rake the hairs on the right side of the mouse's chest to make them stand straight up and trim the hair close to the skin using small scissors. To locate the vein, look closely for jugular vein movement. Using forceps and small scissors, make a small incision from the sternum toward the ear over the vein^11^.
3.1.6.5 Attach the tubing-attached saline syringe to the sterilized catheter to flush and fill it with sterile saline. Leaving them connected, place the syringe and catheter on the platform near the mouse's right shoulder.
3.1.6.6 With hemostats, tunnel through the front incision under the skin to the back incision, opening and closing the hemostats very slightly along the way. Very gently pinch the beveled end of the flushed catheter with the hemostats and pull it through the tunnel to the front of the animal (Figure 4B). Turn the hemostat handle away from you and set it down behind the right shoulder of the mouse. Support the pointed end on a stack of gauze or with the workstation “helping hands” so that the hemostats are pointed slightly upward and hold the catheter tip out of the way.

#### 3.1.7. Identify, Isolate, and Prepare Jugular Vein

3.1.7.1 Small pinching and pulling movements with curved forceps can be used to find the vein, which can often be seen just above the chest muscle wall in the incision area^12^.
3.1.7.2 Once the vein is visible, place forceps in the closed position into the fascia and fatty tissue just to the side of the vein, then allow them to spread open along the vein's length. With the first pair of forceps left in place, put the other set of forceps, closed, into the same opening and perform the same motion from the opposite direction. Repeat this back-and-forth, with the left and right forceps, several times on one side of the vein. Then repeat on the other side, until the vein is isolated and can be lifted.
3.1.7.3 Move fatty tissue attached to the vein by grasping it with forceps (as far from the vein as possible) and pulling parallel to the length of the vein. Be careful not to snag or grab the thin, clear wall of the vein, as this can tear easily, and cause bleeding. Keep the vein hydrated with saline from the rinsing syringe throughout this process.
3.1.7.4 Cradle the isolated vein with forceps (Figure 4C) and carefully slide the short surgical bar under the vein to keep it isolated (Figure 4D).
3.1.7.5 If more substantial tissue strands are still present alongside the vein and are visibly distinguishable from it, use forceps to sever them before moving on.
3.1.7.6 Cut two ~1″ lengths of suture thread. Using forceps, place them under the vein, one at the lower end (below the bar) and one at the upper (above the bar). Turn each thread into a very loose knot around the vein by crossing the ends and pushing one end through the hole twice. Only pull the ends of each knot slightly, so they make relatively wide but secure loops around the vein (Figure 4D).
3.1.7.7 Free the catheter tip so that it drops down toward the vein. Using forceps, gently insert it under the top suture loop, running in the direction of the tail, before re-securing it with the hemostats.
3.1.7.8 Place a stack of gauze next to the head of the mouse to serve as a support for the catheter sled, ensuring that the gauze does not impede the breathing of the mouse.

#### 3.1.8. Make a Veinous Incision and Insert the Catheter

3.1.8.1 Using forceps in your non-dominant hand, grasp the surgical bar; slight upward pressure on the vein is to be maintained to limit bleeding. Be careful not to tear the vein. Open the artery scissors and orient them downward, placing the ball-tip snug to the underside of the vein and on the same side as the heart. Then close the scissors, which will result in a small cut on the top side of the vein.
3.1.8.2 Continuing upward pressure on the vein, clear the area with the rinsing syringe. Use forceps or a needle holder to insert the end of the catheter into the vein incision^13^, pointing toward the heart. A successful vein entry will usually give no resistance^14^.
3.1.8.3 If needed, the tip of the catheter sled <5 mm can be inserted. Rest the sled on the stack of gauze and use it to guide the catheter into the vein^15^.
3.1.8.4 Check that the catheter is in the vein by slowly pulling back on the syringe plunger and watching for blood in the tubing (Figure 4E)^16^. If successful, slowly push the blood back into the animal, using care not to introduce much saline.

#### 3.1.9. Close Veinous and Ventral Incisions

3.1.9.1 Remove the sled, if present, and perform the next steps in quick succession. Relieve the upward pressure on the metal bar, lowering it to rest on the mouse, and tighten the lower and the upper sutures to stop the bleeding^17^.
3.1.9.2 If possible, tie a second double overhand knot for each suture and tighten, pulling evenly on both ends, so that two knots lie flat on either side of the vein. Check the blood flow with the syringe again, then if okay, trim the suture ties to ~2 mm length.
3.1.9.3 Reach behind the animal and pull the catheter base slightly away from the animal to get rid of excess length. Then, using forceps, pinch the skin around the jugular incision, avoiding the catheter. Jiggle the incision up and down gently to settle in the catheter.
3.1.9.4 Clean the incision site using cotton-tipped applicators and saline. Suture together the incision, using a modified simple interrupted stitch.
3.1.9.4.1 Hold together the two sides of the chest incision lengthwise with curved forceps and begin stitching from one end.
3.1.9.4.2 For each stitch, pierce the skin 1–2 mm away from the incision on both sides, running the needle under the pinched-together incision. Using the long end of the suture, loosely wrap the end of the hemostats twice, then use them to grab the short end of the suture and pull both ends until just tightened at the skin.
3.1.9.4.3 Repeat the wrapping of the hemostats, this time in the other direction. Pull each side evenly so that the knots lie on either side of the incision, then trim each end to <2 mm.
3.1.9.4.4 Repeat stitches at close intervals along the length of the incision.
3.1.9.5 Clean the incision area again with saline.

#### 3.1.10. Position the Catheter Base and Close the Dorsal Incision

3.1.10.1 Turn the mouse and nose cone to the prone position, adjust the anesthesia settings if needed, and insert the mesh base of the catheter underneath the skin of the back incision.
3.1.10.2 Turn the catheter so that the tubing is oriented toward the jugular vein. Check for blood flow with the syringe, as before, to be sure that the tubing position does not impede it.
3.1.10.3 Pull the catheter base snug against the posterior end of the incision (Figure 4F) and stitch the anterior end of the incision closed, starting at the catheter, in the manner described previously.

#### 3.1.11. Post-operative Care (Immediate)

3.1.11.1 Using the tubing-connected syringe with cefazolin/heparin solution, push fluid to the tip of the tube, then connect and flush the catheter (0.02–0.03 mL).
3.1.11.2 Inject the mouse subcutaneously on one side of the lower back using a warmed hydration syringe. Apply triple antibiotic cream to both incisions.
3.1.11.3 Holding the catheter base securely, push a plastic cap onto the guide portion of the catheter using a twisting motion, then cover it with a metal dust cap before turning off the anesthetic gas.
3.1.11.4 Move the mouse to the recovery cage. Once the mice are awake and mobile, they can be moved to a clean housing cage with food and water.
3.1.11.5 At this point, the mice can be co-housed with any original cage mates which are also anesthetized on the same day.

#### 3.1.12. Between Surgeries

3.1.12.1 Rinse instruments with water, bead sterilize them, and soak in ethanol. Clean and re-sterilize the surgical area, then move tools to the platform to dry.

#### 3.1.13. Continued Post-operative Care

3.1.13.1 Mice should be checked daily for signs of dehydration, pain, and infection (see below) for at least 8 days following surgery (Days 1–8), and as needed after that.
3.1.13.1.1 **Day 1 (the day after surgery):** Refrain from handling surgery mice unless they meet the health-related criteria described below.
3.1.13.1.2 **Days 2–8:** Flush the catheter daily with cefazolin/heparin solution (0.02–0.03 mL/mouse/day). Cefazolin should be mixed fresh daily (see section 2.2.3). Apply triple antibiotic cream to incisions once daily for at least 3 days, and consider at least one saline injection, as described below, for hydration. The mice that are fully recovered from anesthesia and recovering as expected from surgery (i.e., not having a hunched, unkempt appearance, dehydration, etc.) can be rehoused with all original cage mates.
3.1.13.1.3 **Starting on Day 9:** Catheters should be flushed at least 5 days per week with heparin-saline (0.03 μL). During self-administration, catheters should be flushed both before and after each session. On days with flushing and no testing, once daily is sufficient. After recovery, catheter patency must be checked throughout the study^18^, as well as any time failure is suspected, by flushing it with ketamine/midazolam (15 mg/mL/0.75 mg/mL) solution (0.03 mL). Loss of righting reflex should be observed within 3 s of infusion, and mice not meeting this criterion should be removed from the study or may undergo surgery for new catheter placement.

#### 3.1.14. Post-operative Complications

3.1.14.1 Post-surgery dehydration is common and potentially lethal. Mice suspected of dehydration should be given 0.5–1.0 mL (s.c.) of warmed saline, as described above for surgery, between 1–3 times/day. An animal slow to recover or having a hunched appearance that is not helped by subcutaneous saline should be given ketoprofen daily (1 mg/mL; 0.05 mL/day) until they improve. Triple antibiotic cream should be applied to incisions that are red or oozing. For all these conditions, the facility vet should be consulted as needed.

### 3.2. Operant Experimental Procedures

#### 3.2.1. General Considerations

3.2.1.1 Operant session length typically ranges between 1 and 6 h per day^19^ and 5–7 days a week. Sessions should be conducted at the same time over days.
3.2.1.2 Timestamps for entries into all ports and magazines should be recorded throughout each session by beam break.
3.2.1.3 Reinforcement schedule can range from fixed ratio 1 (FR1), which means that one active response (outside of timeout periods) equals one infusion, to greater FR levels.
3.2.1.4 Experiments typically consist of multiple phases of testing, with each phase running for a various number of days. When deciding which phases to include in each experimental design, limitations in the duration of catheter patency should be a consideration. In this section, we provide general procedural guidelines for running operant sessions. Details and considerations for common specific phases of testing, along with example statistical analysis and results, are included in the Anticipated Results section.

#### 3.2.2. Prior to Testing

3.2.2.1 As handling during flushing is somewhat stressful, additional, non-stressful handling (very similar across test animals) is recommended for at least 3 days leading up to testing.
3.2.2.2 The experiments described here do not require either food restriction or food training, and in fact, are more informative without their use^20^.

#### 3.2.3. Running Operant Sessions

3.2.3.1 Mice should be allowed to acclimate to the testing room in their home cages for 15–60 min before each session.
3.2.3.2 When entering the mouse's body weight, be sure to use the proper unit as defined by the program.
3.2.3.3 Mice should be flushed with heparin-saline before and after each session.
3.2.3.4 All trials should begin with a single infusion “prime” of the drug line (with cue), instigated manually in the box or remotely using the program.
3.2.3.5 Once a trial has ended, mice should be removed from the box as soon as possible and returned to their home cage.
3.2.3.6 Boxes should be cleaned as defined in the relevant animal protocol. Mice may perform operant conditioning better with less stringent (i.e., water only) cleaning of their box between sessions.
3.2.3.7 At least two times per week, drug lines should be sterilized with 70% ethanol (contact time of 1 min), then flushed with sterile water, followed by sterile saline.

#### 3.2.4. Troubleshooting

3.2.4.1 If other magazines or manipulanda are in the testing box, consider covering them with metal sheeting held in place with magnets placed on the outside of the apparatus.
3.2.4.2 A plan for animals that struggle to acquire the task should be made in advance^21^. Options include (1) placing a food reinforcer (such as Ensure®) around the active manipulandum, (2) priming the session (at the beginning or in the middle) with 1–3 infusions manually, (3) increasing the acquisition dose (e.g., 3.2 mg/kg/infusion) for one session, and/or (4) conducting overnight sessions (1–3), which can be very helpful especially for animals tested during the light phase.
3.2.4.3 If catheter patency fails, a mouse may receive another catheter to the other jugular vein, followed by the same recovery postoperative care. Reacquisition (as described in the Anticipated Results section) should be demonstrated before returning the mouse to the unfinished testing phase where they were left off.

## 4. Anticipated Results

### 4.1. Experimental Timeline

**Figure 5:**
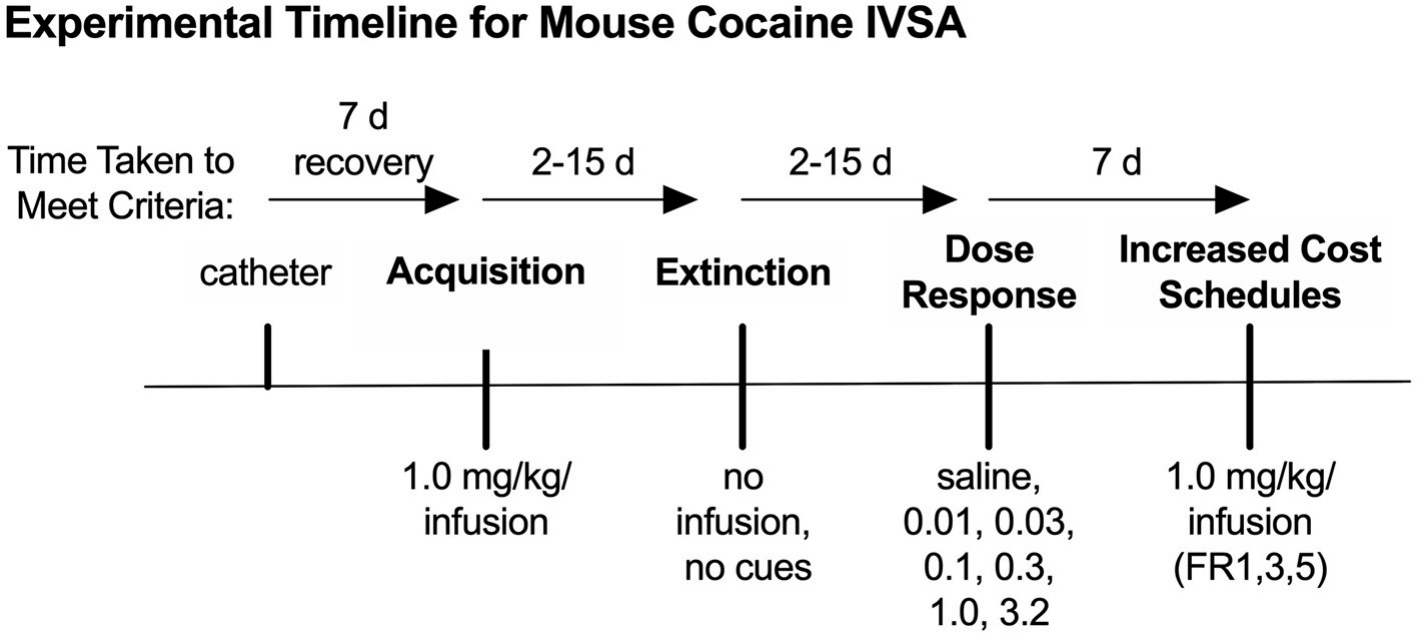
Behavioral timeline for example data. Timeline for the example IVSA experiment, including acquisition, extinction, dose-response, and increased cost schedule phases of testing. Modified from Huebschman et al., 2021, with permission from Wiley and the Federation of European Neuroscience Societies. The figures include data collected with support from the NIDA Drug Supply Program (gifted drug) and Texas A&M University (LS).

Experimental timelines for IVSA studies typically include multiple phases of testing, as mentioned above, which may vary based on the objectives of the experiment. For example, a reinstatement phase will provide insight into drug-seeking behaviors after withdrawal (De Vries et al., 1998), while a dose-response phase will identify changes in sensitivity, tolerance, or hedonic set point (Schenk and Partridge, 1997; Ahmed and Koob, 1998). One possible timeline, including acquisition, extinction, dose-response, and increasing cost schedule phases, is provided as an example in Figure 5. In the following sections, we provide anticipated results (reprinted with permission from Huebschman et al., 2021), discuss advantages, limitations, potential pitfalls, and troubleshooting options for each of these phases. Unless stated otherwise, operant sessions in all phases were 3 h per day and ran 5 days/week. Sessions were terminated early if the reinforcer limit was reached (1.0 mg/kg/inf = 30 max; all lower doses = 100 max; 3.2 mg/kg/inf = 10 max with 10 min timeouts if > 2 reinforcers in <10 min), and all reinforcers were followed by a 20 s time-out period during which additional reinforcers were unavailable.

### 4.2. Acquisition

Acquisition sessions began with the illumination of the active port cue light and a single 1.0 mg/kg infusion of cocaine, after which nose-pokes in the active port resulted in reinforcement (1.0 mg/kg/infusion), as well as cue light illumination, on an FR1 schedule of reinforcement. This dose and schedule were selected to induce stable drug-taking in a large percentage of animals. To limit the contributions of overtraining and variation in total drug intake during this phase, each mouse remained in acquisition only until a predefined set of learning criteria was reached and thus individual animals differed in the number of sessions during this phase. These criteria were defined as two consecutive sessions having ≥15 reinforcers with no more than 20% variation in reinforcers earned between those sessions, and at least 70% discrimination for the active port. If “over-learning” is not a concern for one comparison group, researchers may opt to run acquisition sessions for a set number of days rather than to predefined learning criteria.

**Figure 6:**
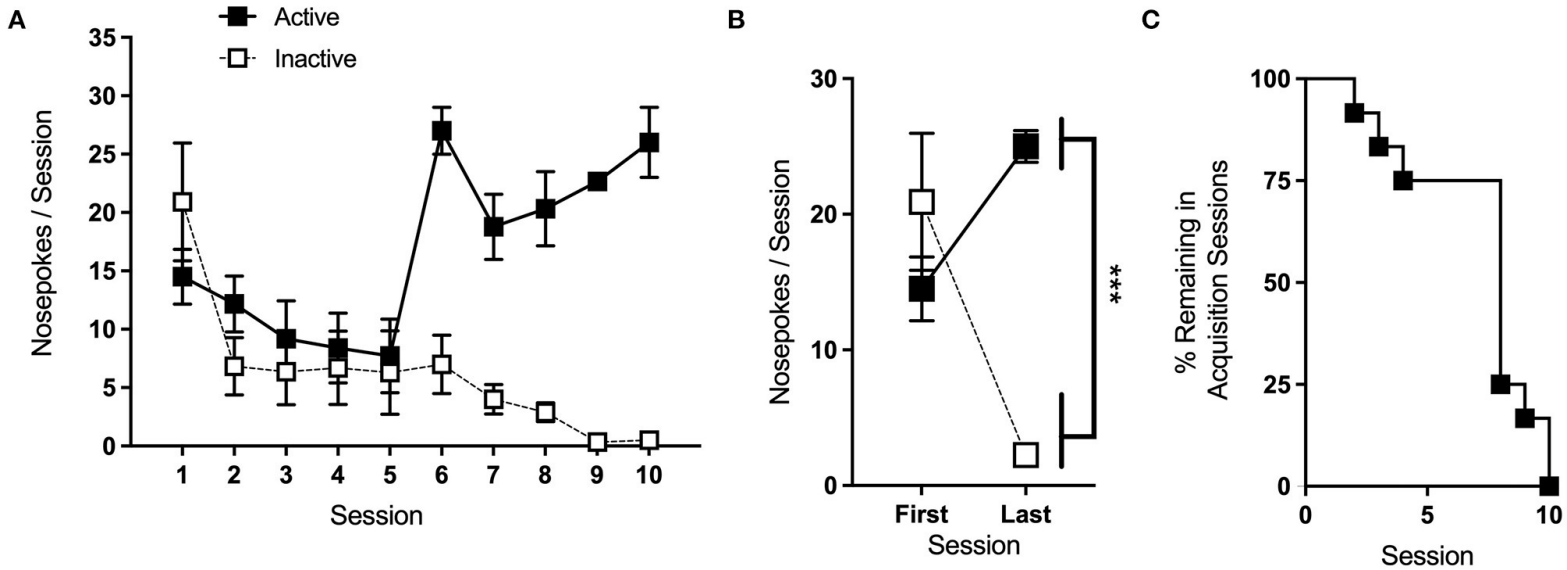
Acquisition. **(A)** Average nose-pokes (active port, large boxes; inactive port, and small boxes), excluding responses made during time-out periods, during acquisition sessions for which >25% of animals remained. **(B)** Mice made significantly more nose-pokes at the active port than the inactive port during the last acquisition session. **(C)** The rate at which animals met the criteria and progressed to the next phase of testing. ***p < 0.001; n/group = 12; data shown are mean ± S.E.M. Modified from Huebschman et al., 2021, with permission from Wiley and the Federation of European Neuroscience Societies. The figures include data collected with support from the NIDA Drug Supply Program (gifted drug) and Texas A&M University (LS).

Average nose-pokes, excluding responses made during time-out periods, for sessions where >25% of animals remained in the acquisition phase are shown in Figure 6A. A two-way repeated measures (RMs) ANOVA comparing nose-poke behavior on each animal's first and last day of acquisition (Figure 6B) reveals a significant interaction of session and port [*F*_(1,11)_ = 26.47, *p* < 0.001]. Follow-up one-way RM ANOVAs show a simple main effect (SME) of port at the last [*F*_(1,11)_ = 234, *p* < 0.001], but not the first, session, with mice making significantly more nose-pokes at the active port than the inactive. SMEs of session were also significant for both the active [*F*_(1,11)_ = 13.3, *p* < 0.01] and the inactive [*F*_(1,11)_ = 13.7, *p* < 0.01] ports, with mice significantly increasing and decreasing in the nose-poke behavior at each port, respectively.

Of the 18 mice included in the acquisition phase, 12 met acquisition criteria within 15 sessions and progressed to later phases of testing. The rate at which these mice met the criteria is shown in Figure 6C. The medium dose used during this phase is intended to maximize the acquisition, and we typically expect ~70% of control animals to meet the criteria. If mice struggle to acquire the task, and it does not interfere with the experimental objectives, interventions as in section 3.2.4.2 can be made to facilitate learning. In the study shown here, overnight sessions were given to animals that had not yet met the criteria after 5 and 10 days.

### 4.3. Extinction

**Figure 7:**
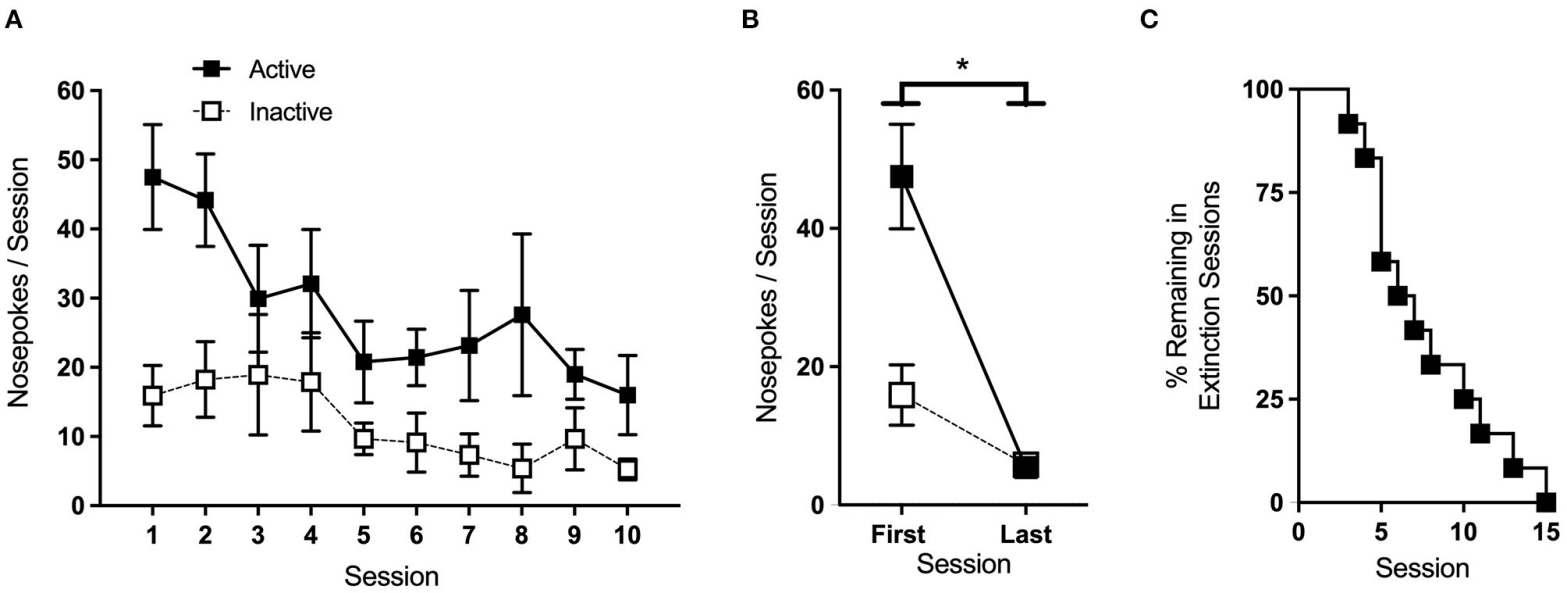
Extinction. **(A)** Average nose-pokes (active port, large boxes; inactive port, and small boxes) during extinction sessions where >25% of animals remained. **(B)** Mice made significantly fewer nose-pokes on the last day of extinction compared to the first. **(C)** The rate at which animals met the criteria and progressed to the next phase of testing. *p < 0.05; n/group = 12; data shown are mean ± SEM. Modified from Huebschman et al., 2021, with permission from Wiley and the Federation of European Neuroscience Societies. The figures include data collected with support from the NIDA Drug Supply Program (gifted drug) and Texas A&M University (LS).

Extinction sessions were conducted in the same manner as acquisition, except that active nose-pokes had no consequence. Animals remained in the extinction phase until active port responding dropped to <30% of the average of the last 2 days of acquisition. Average nose-pokes for sessions where >25% of animals per group remained in the extinction phase are shown in Figure 7A. Two-Way RM ANOVA of nose-poke responses on the first and last day of extinction showed a significant main effect of the session [*F*_(1,11)_ = 5.87, *p* < 0.05], with mice making fewer nose-pokes overall on the last day (Figure 7B). Of the 12 mice that underwent extinction, 2 failed to meet the phase criteria within 15 sessions and were excluded from further analysis. The rate at which the animals met the extinction criteria is shown in Figure 7C.

In this study, active-port nose-pokes during extinction had no consequence. Depending on the objectives of the study, extinction sessions may be run such that active nose-pokes either do or do not trigger cue-light illumination. The former helps ensure that, during drug delivery phases, animals respond to the active port for the reinforcer itself and not to the associated cues. On the other hand, the latter is necessary to examine cue-induced reinstatement after the extinction phase, as it is the reintroduction of the cue-light that drives the reinstatement of drug-seeking behavior. Ideally, in either case, saline replaces drug infusion during extinction, a practice that also maintains any sound cues associated with pump operation. However, if necessary, infusions can be omitted. A small percentage of animals (~15% in our hands) may take 30–45 days to meet the extinction criteria, so it may be beneficial to set a cutoff point at the onset of the study. In the example provided here, we used a cutoff of 15 sessions, and mice that took longer were omitted from the additional testing phases.

### 4.4. Dose-Response and Increasing Cost Schedule

**Figure 8:**
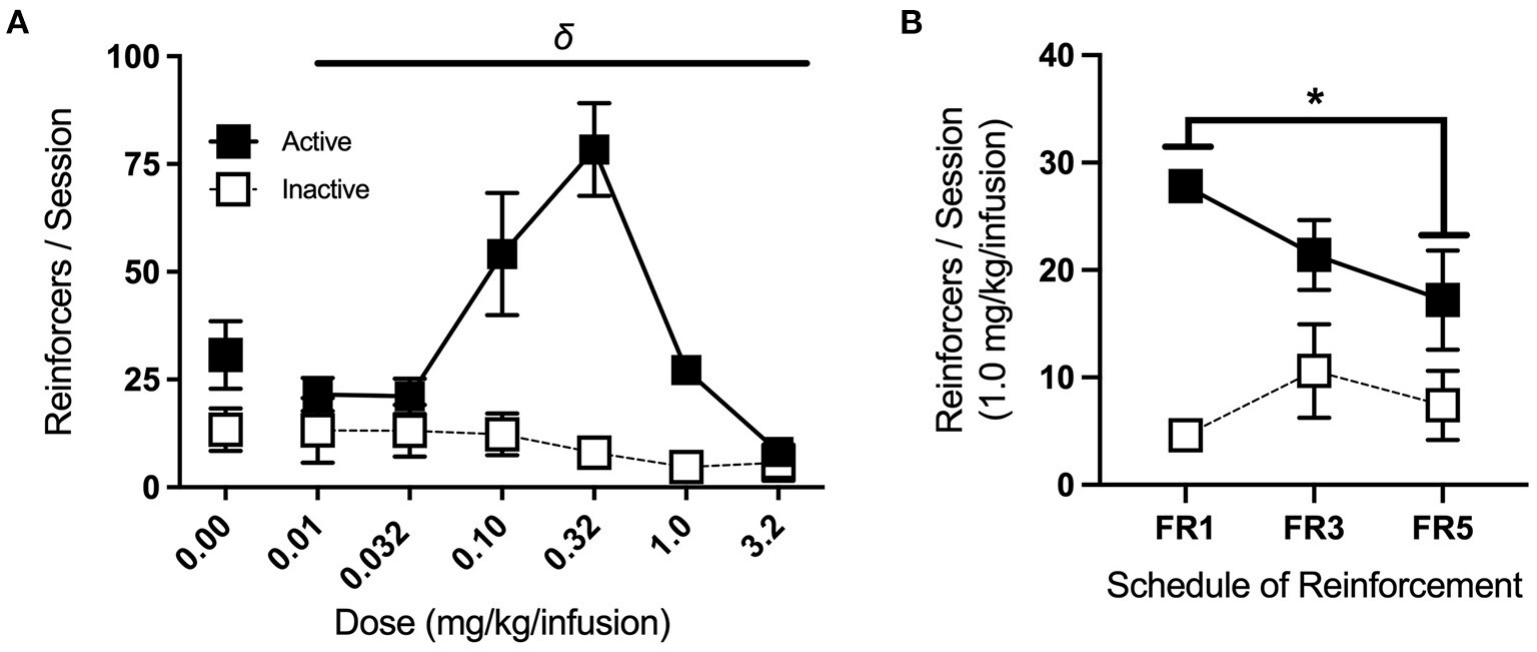
Dose-response and increasing cost schedule. **(A)** Average nose-pokes (active port, large boxes; inactive port, and small boxes), excluding those made during time-out periods, during dose-response testing. There was a significant effect of dose on active port responses (indicated by δ), with responses for the 0.32 mg/kg/infusion dose being significantly greater than those at the 0.01, 0.032, 1.0, and 3.2 mg/kg/infusion doses (n/group = 9). **(B)** Average nose-pokes across increasing schedules of reinforcement for the acquisition dose, with mice making fewer responses at the active port in FR5 sessions compared to FR1 sessions (n/group = 5). ^δ^*p* < 0.001, *p < 0.05; data shown are mean ± SEM. Modified from Huebschman et al., 2021, with permission from Wiley and the Federation of European Neuroscience Societies. The figures include data collected with support from the NIDA Drug Supply Program (gifted drug) and Texas A&M University (LS).

After completing the extinction phase of testing, animals began re-acquisition sessions, which were identical to acquisition sessions and conducted until reinforcers earned in a single session returned to ≥15. All mice met these criteria within 1–2 sessions. For dose-response testing, a single concentration (0, 0.01, 0.032, 0.1, 0.32, 1.0, and 3.2 mg/kg/infusion, in 0.9% saline; see Table 5) was made available for each session. Doses were presented in sequential order, with the starting dose counterbalanced across groups using a Latin square design. Two-way RM ANOVA revealed a significant interaction of dose and port [*F*_(2.11, 12.69)_ = 9.64, *p* < 0.01], with follow up one-way RM ANOVA showing a significant effect of dose for the active port alone [*F*_(2.08, 16.63)_ = 14.9, *p* < 0.001] (Figure 8A). *Post-hoc* Bonferroni pairwise comparison indicates that responses on the active port for the 0.32 dose were significantly higher than those for the 0.01, 0.032, 1.0, and 3.2 mg/kg/infusion doses (*p* < 0.05, 0.05, 0.01, 0.01, respectively) and responses for the 1.0 dose were significantly greater than for the 3.2 dose (*p* < 0.001).

Following dose-response testing, mice returned to the acquisition dose (1.0 mg/kg/infusion) on an FR1, followed by an FR3, and then an FR5 schedule of reinforcement (two consecutive sessions each) (Figure 8B). Three-way RM ANOVA revealed a significant interaction of cost schedule with port responses [*F*_(2,8)_ = 9.42, *p* < 0.01], and follow up one-way RM ANOVA identified a significant effect of cost schedule for active port responses alone [*F*_(2,18)_ = 5.71, *p* < 0.05]. *Post-hoc* Bonferroni pairwise comparison showed a significant difference in active port responses between FR1 and FR5 (*p* < *0.05*), with mice making fewer responses at the higher schedule of reinforcement.

Inverted U-shaped dose-response curves, as seen here, are typical of drug self-administration tasks, as rodents adjust their responses to maintain targeted minimum satiety (Tsibulsky and Norman, 1999). Dose-response testing can provide valuable information, as left or right horizontal shifts in the dose-response curve indicate changes in sensitivity or tolerance (Schenk and Partridge, 1997). On the other hand, vertical shifts have been associated with altered hedonic set points after prior drug experience or escalating drug intake (Ahmed and Koob, 1998). Upward shifts are also observed in animals that display stronger responses on increasing cost schedules of reinforcement (Piazza et al., 2000), so the inclusion of both phases of testing allows for a more robust characterization of behavioral phenotypes.

## 5. Discussion

Intravenous self-administration using operant conditioning techniques has proven a preferred way of studying substance abuse due to its relative resemblance to drug-taking behavior observed in humans. However, amongst rodent animal models, the rat has predominantly been used for IVSA, likely due to technical challenges associated with implanting and maintaining catheters in mice. However, as several labs have demonstrated, the IVSA can be performed in a mouse model and could be more accessible to labs requiring the method. In the Methods section, we describe our optimized approach for conducting cocaine IVSA in mice, including design details for a custom, back-mount catheter that can be prepared in-house, as well as detailed technical surgical advice tailored to jugular vein catheter implantation in this species. We show that these methods result in successful cocaine self-administration using data from the wildtype mouse group in our recently published manuscript, which can be consulted for additional details (Huebschman et al., 2021). In the 2021 study, we moved mice through the acquisition and extinction phases of cocaine IVSA based on when they met the criteria set for learning, then we performed tests for dose-response and increased cost. As expected, wildtype C57BL/6N mice acquired and extinguished the task quickly, showed an inverted u-shaped curve during dose-response testing, and earned fewer reinforcers as schedule requirements were increased from FR1 to FR5.

When we look more closely at our representative results, we see that wildtype mice show a clear preference over the course of acquisition for the active (cocaine + cue light) instead of the inactive (no effect) port. While running each phase to criteria prevents us from cleanly assessing learning over the same number of sessions, mice averaged ~25 active port responses on the second day of meeting criteria requirements. Reflecting this level of intake, mice remaining in sessions six through 10 show, on average, from 20 to greater than 25 active port responses (per 3-h session). These results resemble findings from others testing cocaine IVSA in previously naïve wildtype mice. For example, using the same acquisition dose and schedule, prior work shows wildtype mouse making ~23 active port responses per 3-h session over the 2 days when criteria were met (Thomsen et al., 2009b). We note that mice shown in this study were required to meet (slightly more stringent) acquisition criteria within seven days, while mice shown in our study took up to 10 days. In another study, C57BL/6 wildtype mice at the same acquisition dose and schedule showed between 20 and 25 infusions/90-min session on days of 6–10 of acquisition, though they received three of these shorter sessions/day (van der Veen et al., 2008). C57BL/6J wildtype mice receiving the same 1.0 mg/kg/infusion dose and FR1 schedule responded for around 29 infusions per 3-h sessions at the point of meeting the acquisition criteria (Stoll et al., 2018).

During extinction, we required more stringent criteria (<30% of the averaged last 2 days of acquisition) than some other published studies [e.g., <80% (Thomsen et al., 2009b)]. As such, response levels are difficult to directly compare, but as expected, our mice average lower responses on the last day of extinction. For example, the average active port response number on the last session of our extinction phase was around five, while the above studies showed ~13 (Thomsen et al., 2009b). Also, a study using the extinction criteria of ≤30% of acquisition level compared to ours very closely, showing about seven active port responses at the criteria (Stoll et al., 2018). The authors report around 16–18 sessions on average to extinction criteria for their control groups, while our mice met extinction criteria, on average, in 8 days. The importance of the extinction phase with cues cannot be overemphasized, as it tests whether self-administration can be supported by exposure to a cue that was previously associated with drug delivery. The phenomenon has been highlighted previously, especially in the C57BL/6 strain (Thomsen and Caine, 2011), and others have shown visual stimulus seeking in C57BL/6J mice, including the reinstatement of visual stimulus-seeking even though no drug or food was ever paired with the stimulus (Contet et al., 2010). Drug reinstatement studies, which necessitate extinction without the discriminative cue, should therefore include the criterion of increased responding when cocaine is again made available after extinction (Thomsen and Caine, 2011). Interestingly, many studies have also capitalized on this interesting observation of operant sensation seeking in mice, using it as an additional tool to understand behavioral addictions more broadly (Olsen and Winder, 2009; Dickson and Mittleman, 2020).

Returning to our representative results, our dose-response findings indicate a peak in wildtype mouse responses at the 0.32-unit dose, for which just over 75 responses were averaged in the 3-h session. This finding resembles wildtype mice published previously. For example, prior work shows wildtype C57BL/6 mice made ~69 responses over a 3-h session (Thomsen et al., 2009a), and another showed ~46 responses made over a 1.5-h session (van der Veen et al., 2008); however, the peak of the average response is often alternatively seen at the 0.1 mg/kg/infusion dose, such as in a study reporting ~60 responses in a 3-h session (Schmidt et al., 2011). Not many studies appear to have tested the mice under increased cost conditions comparable to the format and drug used in our example work, but our findings are very similar to our prior published work, where wildtype C57BL/6N mice earned around 19–24 infusions during 3-h sessions testing FR1, FR2, and FR3 schedules at this same cocaine dose (Penrod et al., 2020). These comparisons highlight that our results are within the normal limits found in the literature—or deviate in an expected way, such as due to more stringent extinction criteria—but we stress that a range of results can be observed across the literature for all self-administration phases or tests and may relate to differences in the mouse strain, timeout periods and maximum allowed responses, position and salience of discriminative cues, presence of unintended cues (such as syringe pump sounds), differences in chamber cleaning practices, etc.

The drug self-administration assay has high face validity and is a valuable tool for studying the reinforcing effects of drugs of abuse and substance use disorders. It can be adapted to investigate a wide range of drugs and drug-related behaviors, including initial drug-taking, changes in sensitivity or tolerance, and persistence or reinstatement of drug-seeking. While drug IVSA in mice presents challenges, there are tremendous advantages to being able to use the mouse research model in this assay, including the availability of genetic options, reduced need for the colony and behavioral testing space, lower cage and supply costs, and the ability to maintain continuity with prior or associated studies performed in mice. Here we present an update to an established methodology for mouse IVSA supported by data in wildtype C57BL/6N mice that compares favorably to other published studies. Our hope is that this detailed protocol for catheter construction, surgery, equipment setup, and basic experimental planning may allow for more researchers to bring the advantages of the mouse model to bear on the problem of understanding the substance abuse.

## Data Availability Statement

The original contributions presented in the study are included in the article/supplementary materials, further inquiries can be directed to the corresponding author.

## Ethics Statement

The animal study was reviewed and approved by Texas A&M University Institutional Animal Care and Use Committee.

## Author Contributions

The article was written by GV, JH, EC, CK, and LS. Contributions to the methodology were made by YG, JH, and LS. Surgery photographs were taken by GV. Behavioral data were collected by JH. All authors contributed to the article and approved the submitted version.

## Funding

This work was supported by NIDA (DA051727) and Texas A&M University internal funding.

## Conflict of Interest

The authors declare that the research was conducted in the absence of any commercial or financial relationships that could be construed as a potential conflict of interest.

## Publisher's Note

All claims expressed in this article are solely those of the authors and do not necessarily represent those of their affiliated organizations, or those of the publisher, the editors and the reviewers. Any product that may be evaluated in this article, or claim that may be made by its manufacturer, is not guaranteed or endorsed by the publisher.

## Footnotes

^1^For some mouse strains or ages, 1.2 cm is too long. If the step for pulling back blood with the syringe is consistently improved by less than maximum catheter insertion, cutting catheters to 1.0–1.1 cm length may help.

^2^You may need to calculate the cross-section area of a syringe. Measure across just the open space of the inner syringe at the widest point to find the diameter. Divide the diameter by 2 = radius, multiply the radius by itself, multiply that answer by pi. To check yourself, see your pump manual's Appendix and compare to similar syringes.

^3^This value will be different for different pumps.

^4^Alternatively, use a pipette to measure volume.

^5^While mice can perform operant conditioning with ports or levers, they may learn the task more quickly with ports.

^6^A fixed amount of time following drug delivery (e.g., 3–20 s) when the drug is made unavailable.

^7^Longer timeouts may be considered based on the amount of drug taken within a given timeframe For example, a 10-minute timeout is triggered when mice take two high (3.2 mg/kg/infusion) doses within a 10-minute period.

^8^Susceptibility to overdose varies by mouse strain, and these limits may not be sufficient in all studies, particularly for non-C57BL/6 mice.

^9^Magnetic twist ties can be used to elevate and secure the nose cone in this position.

^10^Make sure that the breathing of the mouse remains stable; if gasping is observed, anesthetic percent and/or flow need to be adjusted downward.

^11^Make sure this incision and the vein are moistened regularly with a sterile saline throughout the duration of the surgery using the prepared rinsing syringe.

^12^If the vein is not visible, forceps can be used to gently slide the top layers of tissue over.

^13^With experience, the catheter may be inserted directly into the vein opening. Look closely for the exit point of blood from the vein when the cut is made will help. If the first few attempts are not successful, targeting the catheter to enter the vein slightly higher than the perceived cut can sometimes increase success.

^14^There are sheaths around the jugular vein that can give the appearance of a successful catheter entry. If the catheter cannot be pushed in fully and produces no blood at pullback, this sheath is often the cause. Double check that the cut made to the vein by the artery scissors resulted in bleeding.

^15^Inserting the sled more than a minimal amount causes rapid death, vulnerability to which differs by mouse size and background strain.

^16^Occasionally, it is necessary to slide the catheter back out of the vein slightly (without removing it) to allow for blood flow, after which the catheter can be pushed back into place.

^17^It is easy to overtighten at this step, so use the syringe to check the blood flow, as described above, afterward. If blood flow is diminished, minimally loosen the ties one at a time and recheck the flow.

^18^Three to four patency check point times should be planned, including just before the first and just after the last sessions.

^19^A major factor in session length is whether escalation in drug-taking will be studied over time, for which 4–6 h sessions have been shown to be required.

^20^Food training may occlude differences in acquisition dose studies, and particularly when no extinction is performed for food training, leaves open the possibility that later responding is driven by food-motivated seeking. Additionally, drug-taking behavior under food-restriction may have limited translational relevance to human drug use.

^21^It is important to either confirm that the need for acquisition interventions does not differ significantly between comparison groups or disclose that it does, if so.
